# Oral phage therapy with microencapsulated phage A221 against *Escherichia coli* infections in weaned piglets

**DOI:** 10.1186/s12917-023-03724-y

**Published:** 2023-09-20

**Authors:** Xinyu Mao, Yuxing Wu, Runwen Ma, Lei Li, Leping Wang, Yizhou Tan, Ziyong Li, Hui Liu, Kaiou Han, Yajie Cao, Yinan Li, Hao Peng, Xun Li, Chuanhuo Hu, Xiaoye Wang

**Affiliations:** 1 College of Animal Science and Technology, Guangxi University, Nanning, 530004 Guangxi P. R. China; 2 Guangxi Zhuang Autonomous Region Engineering Research Center of Veterinary Biologics, Nanning, 530004 Guangxi China; 3 Guangxi Veterinary Research Institute, Nanning, 530004 Guangxi China

**Keywords:** Oral, Microencapsulated phage, Treatment, *Escherichia coli*, Diarrhea

## Abstract

**Background:**

*Escherichia coli (E. coli)* is a common pathogen that often causes diarrhea in piglets. Since bacteria are becoming more and more resistant to antibiotics, phages have become a promising alternative therapy. However, the therapy of oral phage often fails to achieve the desired effect. A novel phage named A221 was isolated by using *E. coli* GXXW-1103 as host strain, characterized by electron microscopy, genomic sequencing and analyzed by measuring lysis ability in vitro.

**Results:**

Phage A221 was identified as a member of *Ackermannviridae*, *Aglimvirinae*, *Agtrevirus* with 153297 bp genome and effectively inhibited bacterial growth in vitro for 16 h. This study was conducted to evaluate the therapeutic effect of oral microencapsulated phage A221 on *E. coli* GXXW-1103 infections in weaned piglets. The protective effect of phage was evaluated by body weight analysis, bacterial load and histopathological changes. The results showed that with the treatment of phage A221, the body weight of piglets increased, the percentage of Enterobacteriaceae in duodenum decreased to 0.64%, the lesions in cecum and duodenum were alleviated, and the bacterial load in the jejunal lymph nodes, cecum and spleen were also significantly different with infected group (*P* < 0.001).

**Conclusions:**

The results showed that phage A221 significantly increased the daily weight gain of piglets, reduced the bacterial load of tissues and the intestinal lesions, achieved the same therapeutic effect as antibiotic Florfenicol. Taken together, oral microencapsulated phage A221 has a good therapeutic effect on bacterial diarrhea of weaned piglets, which provides guidance for the clinical application of phage therapy in the future.

**Supplementary Information:**

The online version contains supplementary material available at 10.1186/s12917-023-03724-y.

## Background

*E. coli* is the most prevalent Gram-negative bacterial pathogen, according to studies, it can cause a wide range of clinical illnesses [1]. It is one of the most important causes of post-weaning diarrhea (PWD) in piglets. Diarrheagenic strains of *E. coli* can be divided into at least six different categories with corresponding distinct pathogenic mechanisms [2]. The enterotoxigenic *E. coli* with F4 and F18 are the two main pathogens associated with PWD in piglets [3]. Antibiotics are the primary antibacterial agents because of their broad spectrum and high efficiency. However, an increasing number of antibiotic resistance genes has been identified in *E. coli* isolates during the last decades [4]. In recent years, antibiotic abuse had caused health emergency situations and had a huge socioeconomic impact [5]. In addition, the use of broad-spectrum antibiotics may cause dysregulation of the gut microbiota, which in turn increases the risk of other bacterial infections [6]. Against the background of this era, phage therapy is being investigated as an alternative therapeutic method for bacterial infections which are multidrug resistance [7].

Phages are the viruses which can infect and replicate within bacteria, and they could be found anywhere, including the water, soil, and air [8]. Garcia, P et al. showed that widely distributed phages can serve as effective biocontrol agents for controlling foodborne pathogens [9]. In fact, there have been multiple studies demonstrating that phages can be used in live animals. El-Gohary, F.A et al. concluded that augmentation of the environment with phage is an effective method to prevent colibacillosis in broiler chickens by monitoring the body weight and mortality of broilers [10]. In a study conducted in 2019, Richards et al. used a phage cocktail containing two virulent Campylobacter phages (CP20 and CP30) to treat broiler chickens colonized with C. *jejuni* HPC5. They found that phages could effectively reduce *Campylobacter* counts in cecum contents without affecting the microbiota structure [11].

The applied dose and preparation that can enable phage to obtain effective antibacterial effect have previously been explored. In a study by Rozema et al., four rectal doses of phage were shown to be less effective against *E. coli* O157:H7 than oral administration [12]. However, the main problem with oral application of phage is the acidity and proteolytic activity in the stomach [13]. In this experiment, we used the sodium alginate to microencapsulated phage A221. Microencapsulation provides a new route for the oral utilization of phage, which can protect them from the gastric environment of the stomach. This helps to ensure that high concentrations of phages reach the actual sites of infection when administered orally [14], greatly improving the availability of phages in clinical medicine.

In this study, we isolated and characterized a phage named A221, microencapsulated it with sodium alginate, and used it for the oral treatment of piglets. This study aimed to investigate the therapeutic effect of oral microencapsulated phage on weaned piglets which was infected with *E. coli*, and provide help for the future clinical application of phage.

## Results

### Isolation and biological characteristics analysis of phage A221

**Fig. 1:**
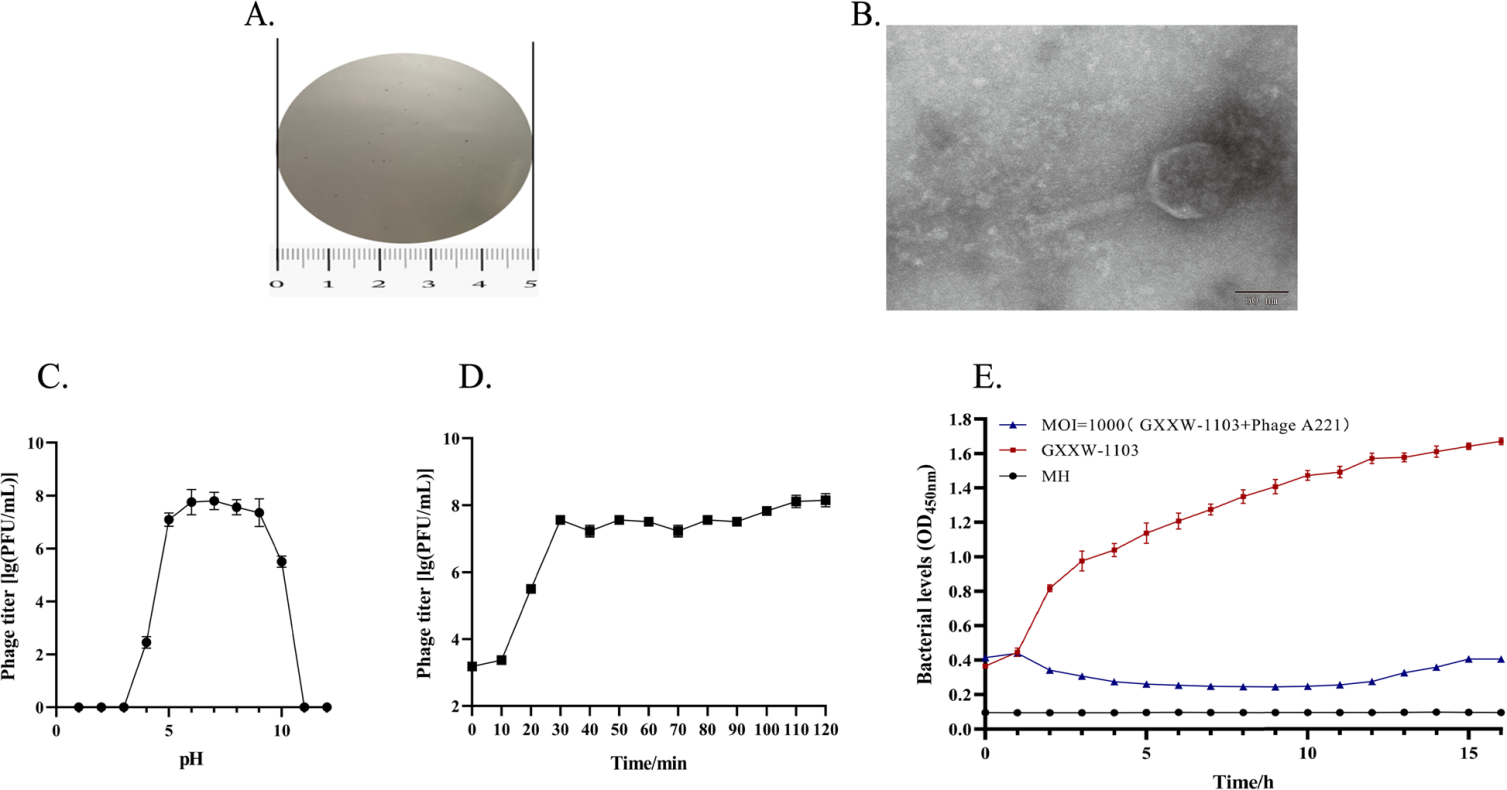
The morphology, biological characteristics and lysis efficacy of phage A221. **A** Plaques of phage A221. Scale in cm. **B** Transmission electron microscopy (TEM) of phage A221. Scale bar 50 nm. **C** Stability of phage A221 in different pH values. **D** One-step growth curve of phage A221. **E** Effect of phage A221 on the growth of *E. coli* GXXW-1103 in broth. All experiments were repeated three times, the error line represents the standard deviation (SD) of the mean

Phage A221 was isolated from pig farm sewage by using *E. coli* GXXW-1103 as host strain. The morphology of phage A221 on double-layer agar plates was presented clear, small plaques in size of 0.2 –0.5 mm in diameter (Fig. 1A). The TEM image showed that phage A221 had a capsid diameter of 90 ± 2 nm and a tail length of 103 ± 2 nm (Fig. 1B).

Phage A221 exhibited stable activity between pH 5.0–9.0 and was inactivated when acid or base levels continued to rise (Fig. 1C). According to the one-step growth curve, the incubation period and burst period of phage A221 was about 10 min and 30 min, respectively (Fig. 1D). The burst size of phage A221 was determined to be 100 ± 2 plaque-forming units (PFU)/cell.

To evaluate the lysis efficacy of phage A221 in vitro, the growth curve of the host strain *E. coli* GXXW-1103 was determined in MH broth. The OD450nm of *E. coli* GXXW-1103 with 1000 MOI phage was maintained below 0.4 during 0 h to 13 h, while the OD_450nm_ of bacteria without phage continuously increased from 0.4 to 1.6. The result indicated that phage A221 had sufficient lysis efficacy in vitro (Fig. 1E).

We determined the host spectrum of phage A221 by using twenty bacterial strains, including fourteen Escherichia coli strains and six Salmonella strains. As shown in Table 1, phage A221 has infectivity on *E. coli* GXXW-1103, FJEC19-2 and SCEC-28. The results showed that phage A221 was a virulent phage with lysis ability against *E. coli* strains, and the host spectrum was not extensive.

**Table 1:** Host strains information of phage A221

Strain type	Strain name	Place	Source	Lysis	Serotype
*Escherichia coli*	CVCC1527	CVCC	Porcine	-	O8: K88
*Escherichia coli*	CVCC4050	CVCC	Unknown	-	O157: H7
*Escherichia coli*	GXEC-11–1	Guangxi	Porcine	-	Undetected
*Escherichia coli*	GXXW-1103	Guangxi	Porcine	+	Undetected
*Escherichia coli*	GXEC-H6	Guangxi	Porcine	-	O86: K61
*Escherichia coli*	GXEC-H7	Guangxi	Porcine	-	Undetected
*Escherichia coli*	GXEC-I11	Guangxi	Porcine	-	Undetected
*Escherichia coli*	GXEC-K5	Guangxi	Porcine	-	O114: K90
*Escherichia coli*	FJEC1-13M	Fujian	Human	-	Undetected
*Escherichia coli*	FJEC19-2	Fujian	Human	+	Undetected
*Escherichia coli*	GDEC17-4	Guangdong	Porcine	-	Undetected
*Escherichia coli*	GDEC-E3	Guangdong	Porcine	-	O127: K63
*Escherichia coli*	SCEC-28	Sichuan	Avian	+	Undetected
*Escherichia coli*	SCEC-E19	Sichuan	Avian	-	Undetected
*Salmonella*	SF-0923	Fujian	Avian	-	*S.* Pullorum
*Salmonella*	SX-1014	Jiangsu	Avian	-	*S.* Pullorum
*Salmonella*	CVCC1806	CVCC	Avian	-	*S.* Enteritidis
*Salmonella*	CVCC3384	CVCC	Porcine	-	*S.* Typhimurium
*Salmonella*	GXSE-S4	Guangxi	Porcine	-	O7, -: Hc: -
*Salmonella*	GXSE-S7	Guangxi	Porcine	-	O2, -: Ha: -

Each strain was tested for three to four replicates

A “ + ” symbolize positive, strain was lysed; A “-” symbolize negative, strain wasn’t lysed

The "Undetected" means the strain was not belong to these serotypes (O157:H7, O157, O114:K90, O126:K71, O26:K60, O142:K86, O127a: K63, O111:K58)

### Genome analysis of phage A221

**Fig. 2:**
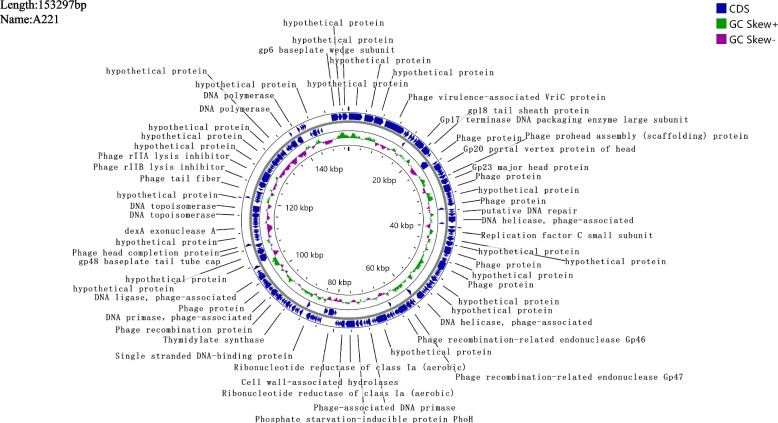
Comparative circular genome map of phage A221. CDS, positive and negative GC are indicated by blue, green and purple, respectively

The length of the whole genome of phage A221(ON862890.1) is 153,297 bp, with a GC content of 49.11%. In the A221 genome, 192 open reading frames (ORFs) were predicted (Fig. 2). No antibiotic resistance and virulence genes were detected in the whole genome of phage A221.

**Fig. 3:**
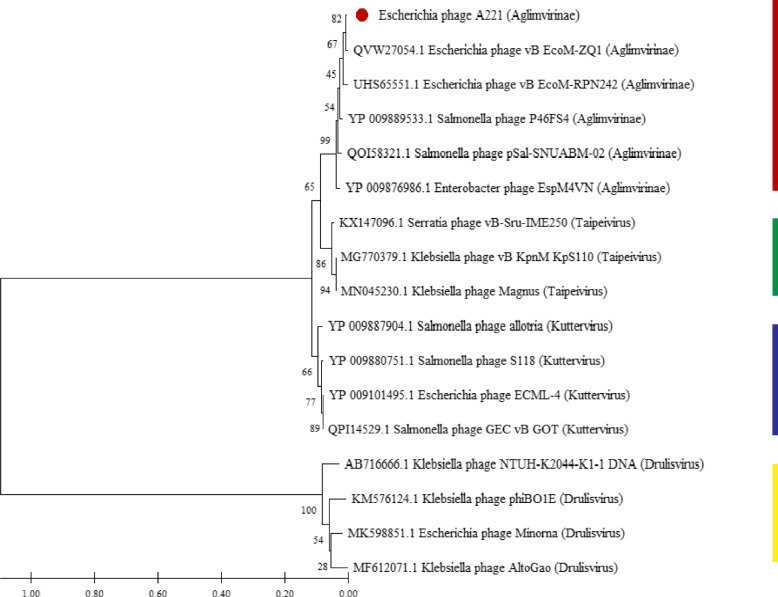
Phylogenetic tree based on DNA polymerase of 16 phages, including phage A221. The tree was generated in Mega 7.0 using neighbor joining method with P distance values and bootstrap replicate of 1000. Phages belong to the Agtrevirus genus, Taipeivirus genus, Kuttervirus genus and Drulisvirus genus are indicated by red line, green line, blue line and yellow line, respectively

DNA polymerase protein sequences were used in a phylogenetic analysis, which revealed that phage A221 belonged to the same branch with Salmonella phage SKML-39 (JX181829.1), Gp43 Shigella phage phiSboM-AG3 (FJ373894.1), Salmonella phage P46FS4 (MT078988.1) and Enterobacter virus phiEM4 (LC373201.1) (Fig. 3). Our data showed that these phages belonged to a same clade and the phage A221 might be a member of that family *Ackermannviridae*, *Aglimvirinae*, *Agtrevirus*.

**Fig. 4:**
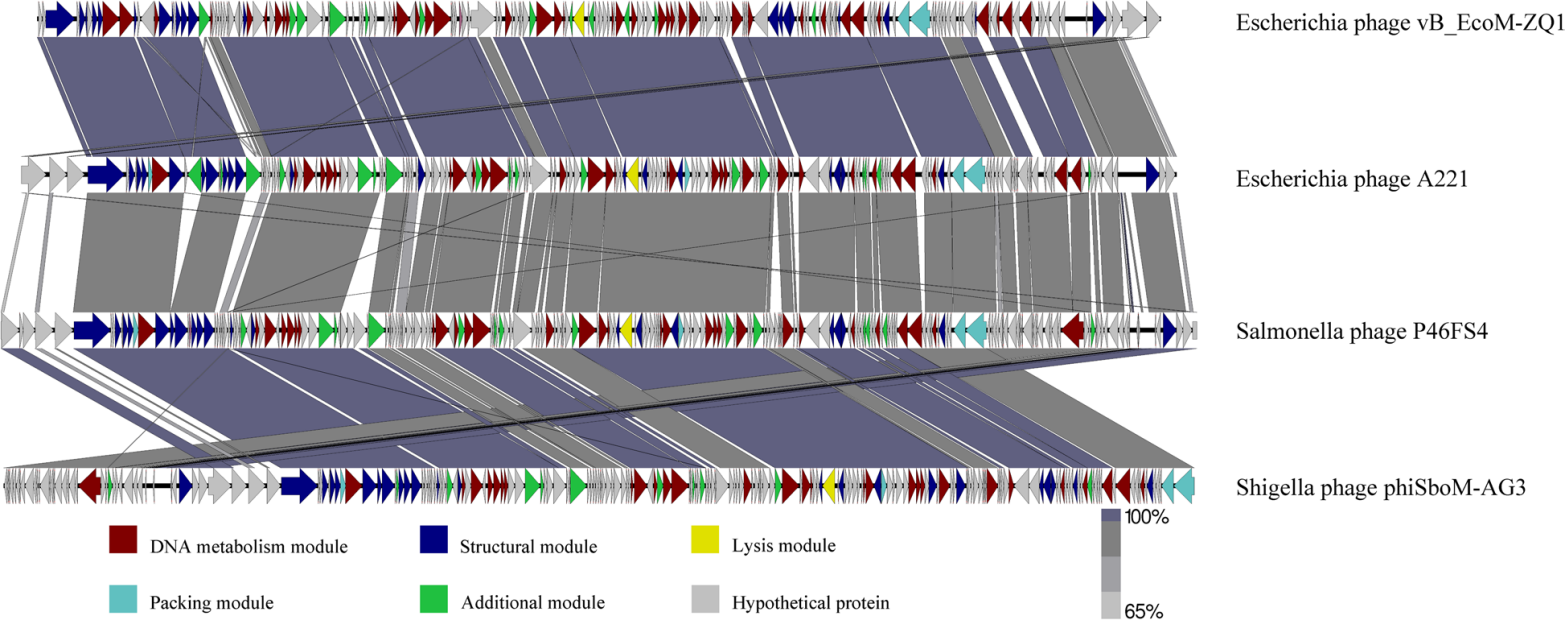
Collinearity analysis of the whole genome of four phages. Collinearity analysis of the whole genome of four phages. According to a previous publication (Botstein, 1980; Nelson, 2004), ORFs can be divided into several modules, including DNA metabolism, lysis, structural, packaging, among others. The results showed that these four phages had high similarity in different modules

For further similarity analysis, phage A221 was compared whole-genome with Escherichia phage vB_EcoM-ZQ1 (MW650886.1), Salmonella phage P46FS4 (NC: 049509.1) and Shigella phage phiSboM-AG3 (NC: 013693.1). The results showed that the four phages had high similarity in different modules (Fig. 4).

### Effect of microencapsulation on phage concentration

The effects of the microencapsulation methods on phage viability were tested. Nontreated (naked) phage produced titers averaging 1.76 × 10^9^ PFU/ml. After dissolved in MBS for 5 min, the titre of the released phages reached 1.39 × 10^9^ PFU/ml, representing a release rate of 78.98%.

### Oral administration of microencapsulated phage A221 reduced *E. coli* infection in weaned piglets

(i) Body weight and average daily weight gain

**Fig. 5:**
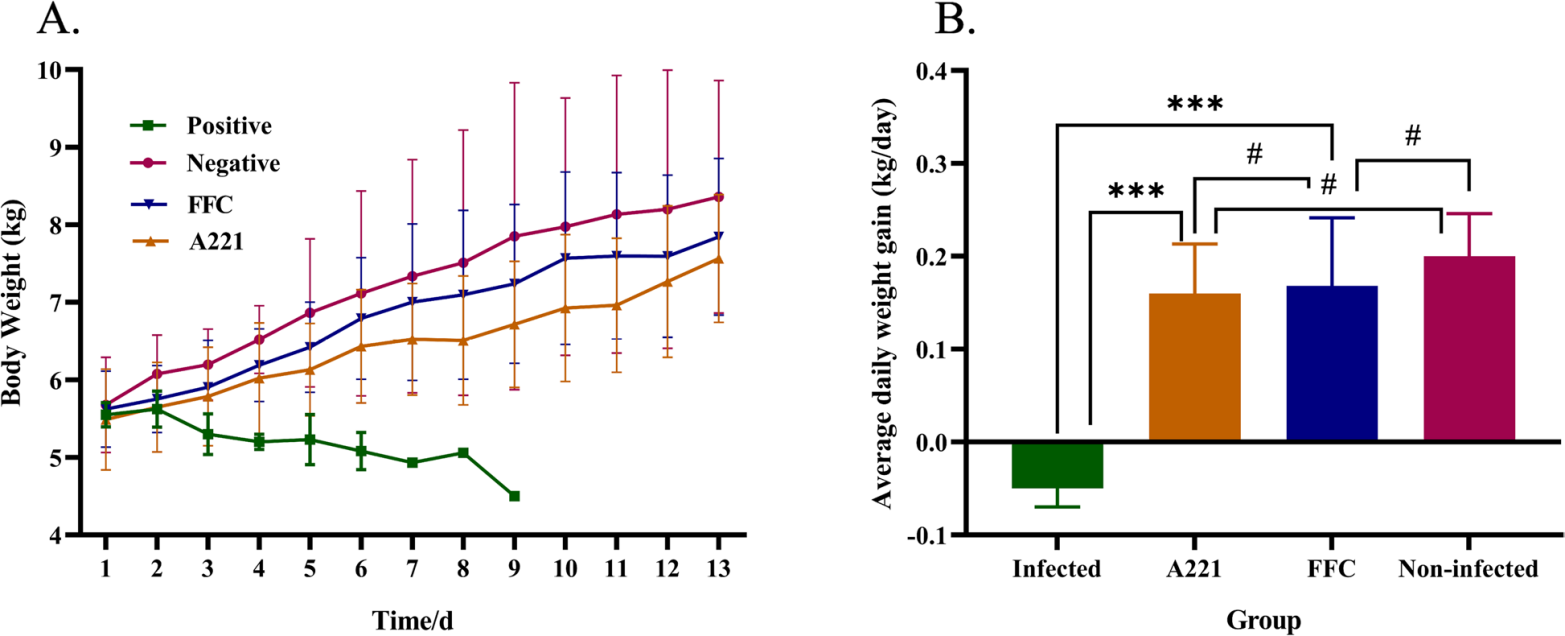
Effect of phage A221 or FFC treatments on body weight. **A** Line chart of body weight in different groups over 13 days; **B** Average daily weight gain in different groups over 13 days. Piglets were infected for the first five days and were treated for the last seven days. The error line represents the standard deviation (SD) of the mean. Statistical analyses were performed with GraphPad Prism 8.0.2 (GraphPad Software, Inc., San Diego, CA, USA) and one-way ANOVA in SPSS (version 23.0) to compare the parameters. “***” bars denote *P* < 0.001. “#” bars denote *P* > 0.05. All experiments were repeated three times

The body weight of weaned piglets was measured to observe the therapeutic effect of phage A221 on piglets. The results showed that piglets in the infected group continued to lose body weight (from 5.6 kg to 4.5 kg) and all piglets died within 9 days, while the body weight of piglets in the A221 group, the FFC group and the non-infected group increased in 13 days, from 5.5 kg to 7.6 kg, 5.6 kg to 7.8 kg and 5.7 kg to 8.4 kg, respectively (Fig. 5A). For further evaluating the growth performance of piglets, we recorded the average daily weight gain of piglets in each group. The average daily gain of piglets in the A221 group was 0.160 kg, which was significantly higher (*P* < 0.001) than that in the infected group (-0.05 kg), no significant difference (*P* > 0.05) compared with FFC group (0.168 kg) and Non-infected group (0.2 kg) (Fig. 5B). These data indicated that oral treatment with microencapsulated phage A221 alleviated the effects of diarrhea caused by *E. coli* GXXW-1103 and restored weight gain in piglets.

(ii) Bacterial loads of tissues and relative abundance of microbiota

**Fig. 6:**
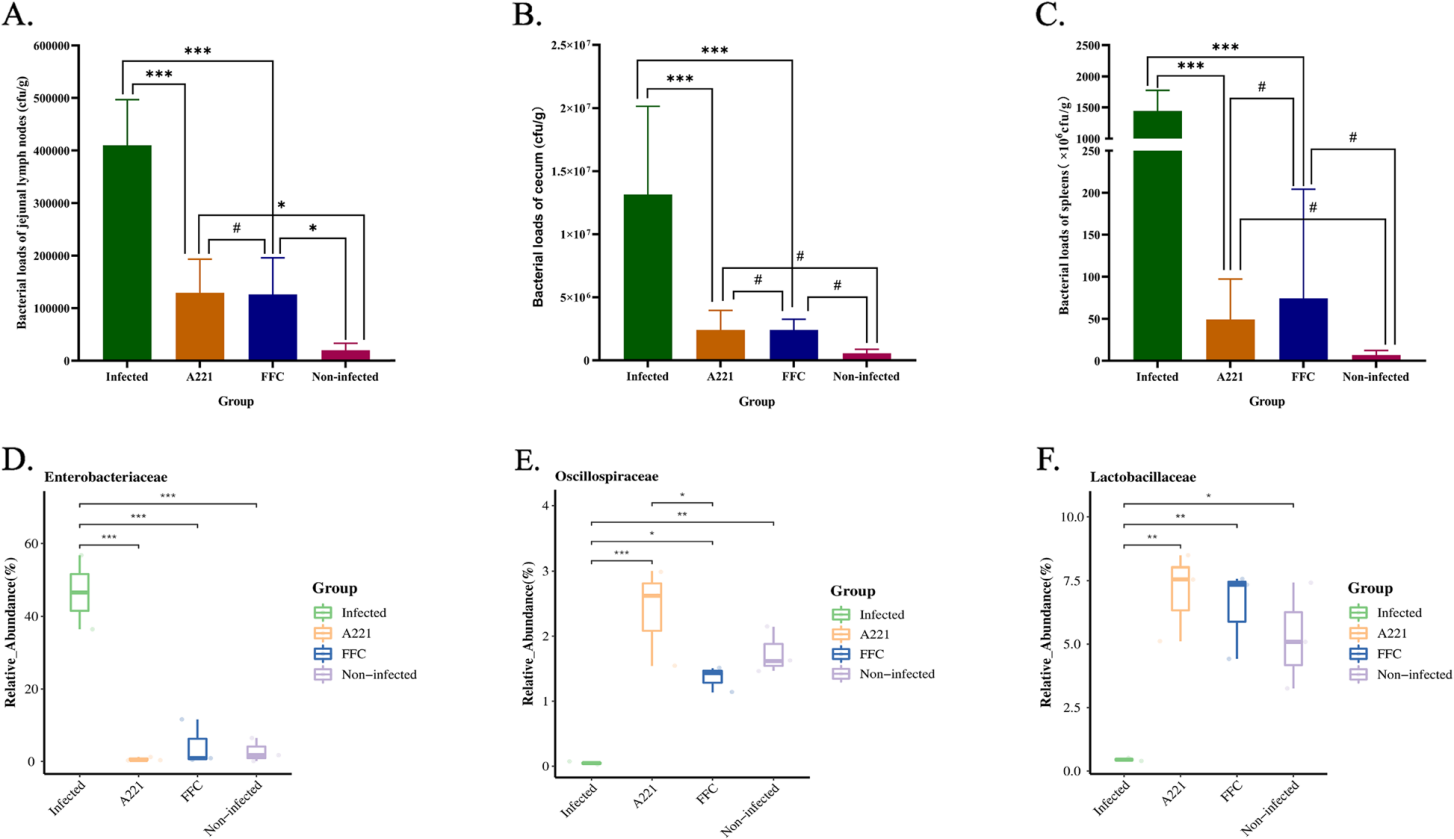
Effects of phage A221 or FFC on bacteria. **A**-**C** The amount of *E. coli* loads in jejunal lymph nodes (**A**), cecum (**B**) and spleens (**C**) of piglets. **D**-**F** The relative abundance of *Enterobacteriaceae* (**D**), *Lactobacillaceae* (**E**), *Oscillospiraceae* (**F**) in the duodenum. The error line represents the standard deviation (SD) of the mean. Statistical analyses were performed with GraphPad Prism 8.0.2 (GraphPad Software, Inc., San Diego, CA, USA) and one-way ANOVA in SPSS (version 23.0) to compare the parameters. “*” bars denote *P* < 0.05, “**” bars denote *P* < 0.01, “***” bars denote *P* < 0.001. “#” bars denote *P* > 0.05. All experiments were repeated three times

The bacterial load in diseased animal tissues is crucial for studying the disease development, so we measured the bacterial load of some tissues of piglets in different groups. The results showed that under the treatment of oral microencapsulated phage A221, the bacterial load in jejunal lymph nodes (Fig. 6A), cecum (Fig. 6B) and spleen decreased (Fig. 6C) significantly (*P* < 0.001) compared with the infected group, and there was no obvious difference (*P* > 0.05) compared with the FFC group. Compared with the non-infected group, the bacterial loads in the cecum (Fig. 6B) and spleen (Fig. 6C) of the A221 group were not significantly different from the negative group (*P* > 0.05), except for the jejunal lymph nodes (*P* < 0.01). Similarly, the proportion of *Enterobacteriaceae* in the intestinal flora of the group A221 was significantly lower (*P* < 0.001) than that of the infected group (Fig. 6D). Furthermore, by comparing the proportions of other bacteria in the duodenum, we found that the proportion of two beneficial bacteria (*Lactobacillusaceae* and *Oscillospiraceae*) in the A221 group was significantly higher (*P* < 0.01 and *P* < 0.001) than their proportions in the infected group (Fig. 6E, F). These results showed, under the treatment of phage A221, the relative abundance of beneficial bacteria in the intestinal flora of piglets was not significantly affected and the number of pathogenic bacteria was significantly reduced.

(iii) Histopathological changes

**Fig. 7:**
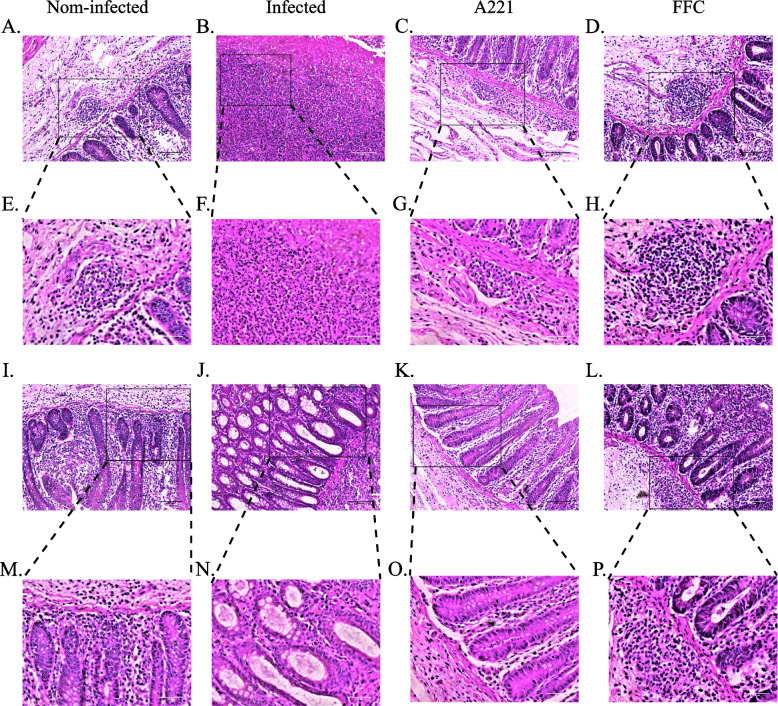
Therapeutic effect of phage A221 or FFC treatments on *E. coli* infection in piglets. Cecum sections of Fig. 7 (**A**-**D** 20 × , **I**-**L** 20 × , **E**–**H** 40 × and **M**-**P** 40 ×) were from piglets sacrificed after 7d treatment (HE stain). Infected piglets were infected with the host bacterial *E. coli* GXXW-1103 and received saline treatment (B + F + J + N), and non-infected piglets received only saline treatment (A + E + L + M). The histopathological changes of piglets on the phage A221 treatment (C + G + K + O) and the FFC treatment (D + H + L + P). Scale bar 150 μm in A-D, I-L and Scale bar 75 μm in **E**–**H**, **M**-**P**

**Fig. 8:**
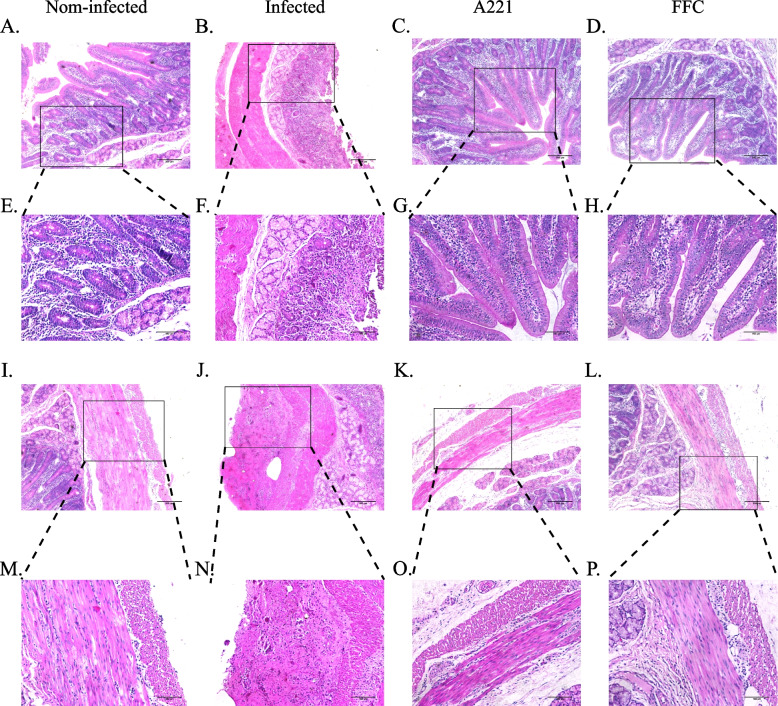
Therapeutic effect of phage A221 or FFC treatments on *E. coli* infection in piglets. Duodenum sections of Fig. 7 (**A**-**D** 20 × , **I**-**L** 20 × , **E**–**H** 40 × and **M**-**P** 40 ×) were from piglets sacrificed after 7d treatment (HE stain). Infected piglets were infected with the host bacterial *E. coli* GXXW-1103 and received saline treatment (B + F + J + N), and non-infected piglets received only saline treatment (A + E + I + M). The histopathological changes of piglets on the phage A221 treatment (C + G + K + O) and the FFC treatment (D + H + L + P)

Pathological sections of the cecum and duodenum were observed to assess the health of the piglets in each group. In the non-infected group, there were no intestinal lesions observed (Figs. 7A, E, I, M and Fig. 8A, E, I, M). In the infected group, caecal lymph nodes were enlarged, intestinal glands were denatured, and only vacuoles were left (Fig. 7B, F, J, N); the intestinal villi of the duodenum were atrophic and degraded, with unclear tissue structure and a lot of bleeding spots in the basal layer (Fig. 8B, F, J, N). In both treatment groups (A221 group and FFC group), intestinal lesions were significantly improved: the size of the lymph nodes in the caecum was similar to the size of the cecum lymph nodes in the non-infected group, the intestinal glands had no obvious lesions (Fig. 7C, D, G, H, K, L, O, P); the intestinal villi of duodenum were intact, with clear tissue structure and a few bleedings points (Fig. 8C, D, G, H, K, L, O, P). These results indicate that oral administration of the microencapsulated phage A221, like oral administration of antibiotic FFC, can reduce the microscopic intestinal lesions in piglets with *E. coli* GXXW-1103.

## Discussion

Weaning factors may affect the immune functions [15] and the intestinal microflora [16] of the piglets negatively. Although the background of post-weaning diarrhea (PWD) should be considered multifactorial, it is often associated with *E. coli*. PWD caused by *E. coli* is a significant cause of economic losses in the pig industry, primarily due to increased mortality and decreased productivity [17]. Piglets with diarrhea often have poor appetite, so when fed antibiotics with mixed feed, the dose of antibiotic will be increased to avoid insufficient dose. This measure has the potential to lead to the abuse of antibiotics and increase bacterial resistance. Unlike antibiotics, small doses of phages can exert curative effects, because it can use host bacteria to replicate themselves in the animal body.

The evolution of bacterial resistance to phages is also under constant investigation. Bull et al. [18] found that phage inhibited bacterial growth in liquid medium for 2-16 h, but the bacterial content slowly increased after too long because some bacteria would develop phage resistance. The study by Lourenço, Marta, et al. [19] research showed that the heterogeneous biogeography of microbes contributes to the long-term coexistence of phages with phage-susceptible bacteria. Nevertheless, there are still more studies that can demonstrate the effectiveness of phage therapy.

In this study, the pathogenic bacteria *E. coli* GXXW-1103 which carried Colistin F18 and Enterotoxin Stb, was isolated from the feces of weaned piglets with severe diarrhea in a pig farm. Colistin F18 adsorbs on the surface of small intestinal epithelial cells, settles, multiplies and then produces enterotoxin, which stimulates the small intestinal epithelial cells to produce a large amount of fluid into the intestinal lumen, causing diarrhea in piglets. Enterotoxin Stb binds to the corresponding receptor, synthesizes cAMP, activates G-regulatory proteins, leads to calcium inward flow, and protein kinase C is activated. Phosphorylated ground protein kinase C causes chloride ions and water to enter the intestinal lumen, leading to diarrhea. The phage A221 was isolated from sewage by using *E. coli* GXXW-1103 as host bacteria. Although the phage A221 cannot lyse many bacteria, it is the only phage isolated in our laboratory that can lyse *E. coli* GXXW-1103 currently. And it does not carry any virulence factor and has sufficient antimicrobial action in vitro.

We tried to treat diarrhea in weaned piglets by using oral phage. However, gastric acid, digestive enzymes, and bile salts (BSs) are highly susceptible to destroying phage, thereby depriving them of their antibacterial activity [20]. The biological characteristics of phage A221 also confirmed its rapid inactivation under gastric acid conditions, so we used micro—capsulate technology with sodium alginate. Microencapsulation technology is a new technology that has developed and matured showing wide application potential. Extending the storage time of active substances and improving their stability can be achieved by using microencapsulation technology [21]. Sodium alginate was normal material used by microencapsulation with the properties of processible, biocompatible, biodegradable and nontoxic for body. Ma et al. [22] investigated in vitro the effect of simulated gastric fluid, bile and intestinal fluid of free phages and phages encapsulated with chitosan-alginate-CaCl2 system, and showed that the encapsulation technique allows most of the phages to remain biologically active in the simulated intestinal environment. Colom et al. [23] compared the retention in the chicken caecum in vivo of orally administered sodium alginate/CaCO3-coated phages and free phages. The results showed that the retention rates of encapsulated phage were higher than those of unencapsulated phage after 2 h, 48 h and 72 h, and the differences were significant (*P* < 0.05, *P* < 0.001, *P* < 0.001, respectively). Both of these studies demonstrated that microencapsulation significantly improved the survival rate of the phages in the gastrointestinal tract compared with free phages. In this experiment, to validate the efficacy of oral administration of microencapsulated phage A221, we established an oral FFC treatment group as a reference for evaluation.

The daily gain can reflect the growth performance of piglets. In this study, under the treatment of oral microencapsulated phage A221, the average daily gain of piglets was significantly higher than that of the infected group (*P* < 0.001), and the weight of piglets showed an increasing trend. A similar study found that weight gain and feed conversion ratios were significantly better in the Clostridium perfringens-challenged chickens treated with multivalent phage cocktail INT-401 than in the Clostridium perfringens-challenged, phage-untreated control birds [24]. These results indicated that phage could reduce the loss of nutrients caused by pathogenic bacteria and restore the absorption and transformation of nutrients. At the same time, Wall et al. showed that using the phage cocktail can reduce the concentration of *Salmonella* on cecum (95%; *p* < 0.05) [25]. In the present experiment, our results agree with previous study, phage A221 effectively reduced the bacterial load in the jejunal lymph node, cecum and spleen of piglets (*P* < 0.001), reaching the same therapeutic level as antibiotic FFC.

The abundance and diversity of intestinal microbiota were closely related to growth performance and post-weaning diarrhea of piglets [26]. It has been reported that antibiotic treatment can shift the population structure of the microbiota and alter bacterial physiology, such as reduction of the amount and diversity of microbes, losses in the function of metabolism and the modulation of the immune system [27]. Conversely, phage can be used to precisely control harmful bacteria and avoid adverse effects like broad-spectrum antibiotic on beneficial bacteria [28]. In this experiment, compared with the infected group, phage accurately reduced the number of *Enterobacteriaceae* in duodenum; compared with FFC group, the advantages of phage specificity and targeting were not well demonstrated. The idea that phages could help rebuild a healthier gut microbiome after treating gut diseases compared to antibiotics needs to be explored with more experiments in the future.

The intestinal barrier is the key structure to protect against parenteral bacterial infection, an intact intestinal barrier is essential in maintaining health [29]. To investigate the pathological changes in the intestines of piglets across different groups, cecum and duodenum tissues were sliced and Hematoxylin–Eosin (H&E) stained. The results showed that *E. coli* GXXW-1103 caused damage to the gut villi and basal layer of cecum and duodenum tissues compared with the non-infected group, while the A221 group and the FFC groups did not exhibit significant intestinal tissue damage. This is similar to a previous study in an enteric murine model, which results showed that phage cocktail targeting *E. coli* O157:H7 has comparable efficacy compared with Enrofloxacin [30]. Our results indicate that oral administration of microencapsulated phage A221attenuates the damage to intestinal integrity by bacterial.

## Conclusion

In summary, oral administration of microencapsulated phage A221 was effective in treating diarrhea caused by *E. coli* GXXW-1103 in weaned piglets. In addition, this treatment can not only achieve the same efficacy as antibiotic FFC, but also effectively avoid affecting other microorganisms in the intestinal flora of piglets due to its targeting of pathogenic bacteria. Combined with the fact that there is no known toxicity, phage therapy against *E. coli* infection in piglets shows great potential. In the future, we intend to explore how to make piglets ingest phage by drinking water, also, although we did not find any harmful effects of phage therapy on the health of piglets in this experiment, we will pay more attention to characterize any possible immune response of pigs to phages in the future research, as has been reported in other animals.

## Material and methods

### Bacteria strains, phage, antibiotics and animal

*E. coli* GXXW-1103, was used for the challenge experiments. This strain was isolated from diarrhea-piglet sources at the farm in Guangxi on 03.11.2020. Take an appropriate amount of fresh bacterial solution, extract the DNA of GXXW-1103 by using the bacterial DNA extraction kit. Referring to the method of Sanches et al. [31]. Amplification of 8 virulence factors such as K88, K99, Stx1, Stx2, F18, Stb, LT and 987P, primer information is given in Table S1. Amplification was performed in a 20 μL reaction mixture containing 10 μL of 2 × green tap PCR MasterMix, 1 ng of prepared template DNA, 1 μL of 10 μM/L forward primer, 1 μL of 10 μM/L reverse primer and 6 μL of ddH_2_O. The amplification program was set as follows: pre-denaturation at 94℃ for 2 min; denaturation at 94℃ for 1 min, annealing temperature for 40 s (set the gradient annealing temperature of 50–62), extension at 72℃ for 30 s (30 × of the above cycle); final extension at 72℃ for 7 min and then stored at 4℃. The PCR products were detected by 1.2% agarose gel electrophoresis, the results showed GXXW-1103 carried in Colistin F18 and Enterotoxin Stb (Fig. S1). This bacterium was kept at − 80°C in 30% v/v glycerol, and saved in clinical veterinary laboratory of Guangxi University.

A phage named A221 was isolated from sewage at a pig farm in Nanning, China, on 14.5.2021. *E. coli* GXXW-1103 was used for phage propagation and plaque counting. Fifty milliliter sewage was centrifuged at 5000 × for 10 min to remove debris pellet and this was repeated 3 times. 3 ml of 1 × 10^8^ CFU/ml *E. coli* GXXW-1103 was mixed with 20 ml of the supernatant after centrifugation and incubated at 180 × g (37℃) for 18 h. In order to remove bacteria and leave phage, the resulting mixture was centrifuged at 10,000 × g for 5 min, and filtered with a 0.22 μm filter. Phage were detected by the conventional double-layered agar method and chosen a single phage plaque for further purification and amplification. The steps for phage purification were repeated three times before all plaques displayed the same morphology. The fltrate which we can utilize to determine plaque morphology and plaqueforming units (PFUs) by the double-layered agar method. Diluted it in SM buffer (5.8 g of NaCl, 2.0 g of MgSO4·7H2O, 50 mL of Tris–HCl [pH 7.4], 5.0 ml of 2% gelatin). After being mixed with soft agar (LB broth,30 g and agar, 0.6% per liter) containing 100 μL of E. coli GXXW-1103 pour the fltrate onto the LB agar plates. The soft-agar overlaid plates were incubated at 37 °C for 12 h to count phage plaques which were estimated as a PFU.

The phage isolated and purified was named A221. For additional research, purified phages were kept in 20% glycerol at -80℃. Before administration, phage A221 was microencapsulated using a previously published protocol [14]. The preparation of phage microcapsules in this study was divided into two steps. The method has been modified as follows. First, accurately weigh 2.2 g of sodium alginate dissolved in 100 ml, 50 mM Tris–HCL (pH 7.5) solution, then add the appropriate amount of phage A221 suspension, stirring and mixing, the final concentration of phage is about 10^8^ PFU/ml. The sodium alginate solution containing phage was dropped into calcium chloride solution to form calcium alginate microspheres, and then put into 0.4% (w/v) chitosan solution (pH 5.0) for encapsulation reaction (20 min at room temperature). The microcapsules were collected, washed and soaked with deionized water, stored at 4 °C.The soluble powder of FFC was procured from Qilu Animal Health Products Co., Ltd. and dissolved by using sterile water. Freshly prepared with sterile water, filtered with the pore size of 0.22 μm before use.

Purchased the experimental animals from Nanning Fulu Farming Co., Ltd. 21-day-old piglets (*n* = 18, half female and half male, weighing 4.61–6.31 kg) were used in this study and were allowed to acclimatize for 1 day before commencement of experiments. These animals were fed a standard non-medicated ration for post- weaning pigs and had water ad libitum and maintained on a 12 h light/dark cycle.

### Determination of host range of phage A221

Host range analysis of phage A221 was determined by the double-layer agar plate method [32]. In short,100 μL of overnight cultures of bacteria were taken and uniformly coated on LB medium plates (Tryptone 10 g/L; Yeast extract 5 g/L; NaCl 10 g/L). Then 5 μL of phage suspension (10^9^ PFU/ml) were apply dropwise to the surface of bacterial plate and saline was added dropwise as a control, incubated at 37 °C for 16–24 h. Clear and transparent lysis zone was produced as the judging standard. The test was repeated three times.

### Transmission electron microscopy (TEM)

Used the transmission electron microscope to analyze the morphology of phage A221 [33]. 50 ml of the phage lysate (approximately 10^9^ PFU/ml) was centrifuged (12,000 g in 20 min), filtered (0.22 μm pore filter) and resuspended in 0.1 mol/L ammonium acetate. The copper grid for TEM was immersed in phage A221 suspension for 10 min to deposit phage on the copper mesh, stained with 2% phosphotungstic acid (PTA, 2% w/v) and dried. After this, the observed phage under a HITACHI HT-7700 transmission electron microscope (TEM, Hitachi High-Tech Co., Ltd, Tokyo, Japan).

### Stability of phage A221 at different pH values and one-step growth

The pH stability of phage ZH4 were evaluated using previously methods [34]. For pH stability testing, filtered high-titer phage A221 was mixed in a series of tubes containing SM buffer of different pH values (2.0–11.0, adjusted using NaOH or HCl), final concentration of phage was 10^9^ PFU/ml, incubated for 2 h at 37 ◦C, and then titer by the double-layer agar plate method to determine the final concentration. The tests were repeated three times. One-step growth experiments were carried out by a modification of methods described elsewhere [35]. 100 μL of phage A221 (1 × 10^7^ PFU/ml) and GXXW-1103 (1 × 10^5^ CFU/ml) were mixed with 3 ml LB broth and incubated at 37 °C warm bath for 15 min, centrifuged at 12,000 rpm for 1 min and discard the supernatant. Then, the precipitate was suspended in LB broth which had been pre-warmed to 37 ℃, followed by incubation at 37 ℃. 100 μL of samples were taken at 10 min intervals (up to 120 min) and immediately diluted, and phage titers were then determined by the double-layered agar plate method. The calculation formula for burst size was: Burst size = phage titer at the end of lysis/number of host strains at the beginning of infection [36]. The tests were repeated three times.

### Extraction of DNA and genome sequencing

Phage genomic DNA was extracted using a previously described method [36]. After the phage pellet was suspended in SM buffer, 20 μL 10% [w/v] SDS, 20 μL 0.5 M EDTA, and 2.5 μL 20 ng/ml proteinase K (P1120, Solarbio, Beijing, China) were added and the mixture incubated at 65℃ for 1 h. An equal volume of phenol–chloroform (1:1) was added to remove the proteinaceous material. The extraction was repeated twice, and the DNA was precipitated according to ethanol precipitation procedures. The pellet was dissolved in 30 μL of distilled water, and the isolated nucleic acids were separated using 0.8% agarose gel electrophoresis, stained with ethidium bromide, and analyzed under ultraviolet (UV) light. After digestion, electrophoresis of the samples in 0.8% agarose containing ethidium bromide (1 μg/ml) was performed.

The construction of sequencing library and the control of sequencing data quality were performed by Personalbio (Shanghai Personal Biotechnology Co., Ltd., China). The tools of genome visualization chose the CGView Server and Easyfig_2.2.5_win. Comparative circular genome map of phage A221 genome was depicted by CG View Server [37].

In order to determine the similarity of A221 with other phages, the genomic sequence of A221 was used to perform the nucleotide BLAST program in NCBI. After that, similar phage genomes were downloaded from NCBI to determine the average nucleotide identity (ANI) using the Mummer alignment tool (ANIm). To further compare the genome similarity, phage genomes similar to A221 were selected for global genome comparison. Linear comparison figure of multiple genomic loci was created by Easy-fig_2.2.5_win based on BLAST [38].

The phylogenetic tree of the phage A221 was constructed based on DNA polymerase protein sequences by MEGA-7. After genome-wide blast, we selected homologous, different genera of the same family, and different families to construct a neighborhood evolutionary tree. The phylogenetic tree was generated in MEGA-7 using neighbor joining method with P distance values and bootstrap replicate of 1000 [39].

### Effect of phage A221 on the growth of *E. coli* in vitro

The Infectivity of phage A221 was measured by bacterial growth in broth. Briefly, 100 μl *E. coli* GXXW-1103 (10^6^ CFU/ml) and 100 μL of phage suspension (10^9^ PFU/ml, 10^7^PFU/ml and 10^5^PFU/ml, separately) were mixed in the 96-well plate. MOI of different groups were 1000, 10 and 0.1. Separate Mueller–Hinton (MH) broth was used as negative control, and *E. coli* GXXW-1103 mixed MH broth was used as positive control. Then the mixtures were incubated with shaking at 180 rpm and 37℃. The ability of phage to inhibit the growth of strain was shown by measuring the OD_450nm_ of the mixture every hour for 16 h at 37 ℃. This assay was performed in triplicate.

### Determination of phage potency after microencapsulated

Prepared Microsphere-broken solution (MBS). Weighed 14.7 g Trisodium citrate dihydrate (final concentration of 50 mM) and 16.8 g NaHCO3 (final concentration of 200 mM), dissolved in 1 L of SM buffer and filtered (0.22 μm pore filter). Take 1 g of microencapsulated phage, put into 9 ml of MBS, lysed at room temperature for 5 min. Detected the PFUs by the double-layered agar method in the cracking solution to calculate the potency of the phage encapsulated in microspheres. Repeat the test three times.

### Animal experiments

Eighteen weaned piglets were divided into 4 groups: 3 piglets for *E. coli* GXXW-1103 infected and with saline treatment (Infected); 6 piglets for *E. coli* GXXW-1103 infected and with microencapsulated phage A221 treatment (A221); 6 piglets for *E. coli* GXXW-1103 infected and with antibiotics Florfenicol (FFC) treatment (FFC); 3 piglets without infection and with saline treatment (Non-infected). All piglets were orally infected with 3 ml of 10^6^ CFU/ml *E. coli* GXXW-1103 per day during days 2–5, except for non-infected group (all bacterial broths were freshly prepared the day before use, the OD of the bacterial broths was measured using a spectrophotometer to standardize the concentration). During the next seven days, the piglets were orally treated with phage A221 (10^9^ PFU/ml, 5 ml) once a day in the phage group (A221 group) and were orally treated with antibiotics FFC (0.14 g/kg) twice a day in antibiotics group (FFC group). After 7 days of treatment, the piglets were euthanized and necropsied. In order to give maximum welfare to the experimental animals, we chose to anesthetize the experimental piglets with sodium pentobarbital at a dose of 100 mg/kg within 30 min and executed by severing the head. The tissues including spleen, caecum and jejunal lymph nodes were harvested for bacterial counts. The collected tissues were weighed and homogenized, diluted in a tenfold gradient, then evenly coated on EMB medium (Eosin-Methylene Blue medium) for incubation, and the number of *E. coli* was determined by taking the plate viable bacteria counting method, each dilution was coated three times. Parts of duodenum and caecum in every group were fixed in neutral buffered formalin and sent to Wuhan Servicebio Technology Co., Ltd. (Wuhan, China) for histological analysis. In addition, duodenal contents from each piglet were collected in 1.5 ml sterile polypropylene tubes, immediately frozen in liquid nitrogen and then stored at -80°C until sent to Shanghai OE Biotech Co., Ltd. (Shanghai, China) for analysis of intestinal flora, which focuses on the analysis of the relative abundance of different flora. All of the experiments were performed according to the guidelines of the regional Animal Ethics Committee and the rules for experimental animals of Guangxi University (GXU-2021–147 and GXU-2021–148).

### Statistical analysis

Using GraphPad Prism 8.0.2 (GraphPad Software, Inc., San Diego, CA, USA) to complete statistical graphs of data. And statistical analyses were performed with one-way ANOVA in SPSS (version 23.0) to compare the parameters. All the experiments were conducted in triplicates. The level of significance was set at *p* ≤ 0.05.

### Statistical analysis

For statistical analyses, it was performed with GraphPad Prism 8.0.2 (GraphPad Software, Inc., San Diego, CA, USA). The significance of the experimental data was determined by multiple T-tests. The error line represents the standard deviation (SD) of the mean. All the experiments were conducted in triplicates.

### Supplementary Information

**Additional file 1:** **Table S1.** Information of primer.

**Additional file 2**: **Fig. S1.** PCR amplified of virulence gene (M, DL2000 DNA Marker;1, *E. coli*-K88;2, *E. coli*-K99;3,* E. coli*-Stx1;4,* E. coli*-Stx2;5,* E. coli*-F18;6,* E. coli*-Stb;7,* E. coli*-LT;8,* E. coli*-987P; -, Negative).

## Data Availability

The genome sequence data of phage A221 has been submitted to the National Center for Biotechnology Information (NCBI) (https://www.ncbi.nlm.nih.gov/nuccore/) under accession number ON862890.1
