# Supplementary material for: Oral phage therapy with microencapsulated phage A221 against Escherichia coli infections in weaned piglets
Source: BMC Vet Res. 2023 Sep 20;19:165. doi: 10.1186/s12917-023-03724-y (PMC10510151; doi:10.1186/s12917-023-03724-y)
Supplement: Supplementary file 1 — Additional file 1: Table S1. Information of primer. [file 12917_2023_3724_MOESM1_ESM.docx]

**Table S1** Information of primer.

| Number | Name | Primer sequence (5′→3′) | | Length | | Temperature |
| --- | --- | --- | --- | --- | --- | --- |
| 1 | *E. coli*-K88 | | F-GGTGATTTCAATGGTTCGGTC  R-ATTGCTACGTTCAGCGGAGCG | | 764 | 62 |
| 2 | *E. coli*-K99 | | F- TATTATCTTAGGTGGTATGG  R- GGTATCCTTTAGCAGCAGTATTTC | | 314 | 50 |
| 3 | *E. coli*-Stx1 | | F- CGATGTTACGGTTTGTTACTGTGACAGC  R- AATGCCACGCTTCCCAGAATTG | | 244 | 62 |
| 4 | *E. coli*-Stx2 | | F- GTTTTGACCATCTTCGTCTGATTATTGAG  R-AGCGTAAGGCTTCTGCTGTGAC | | 324 | 62 |
| 5 | *E. coli*-F18 | | F- GTGAAAAGACTAGTGTTTATTTC  R- CTTGTAAGTAACCGCGTAAGC | | 510 | 50 |
| 6 | *E. coli*-Stb | | F- TGCCTATGCATCTACACAATC  R- GCAGTGAGAAATGGACAATG | | 283 | 55 |
| 7 | *E. coli*-LT | | F- CGGCGTTACTATCCTCTCTA  R- ATTGGGGGTTTTATTATTCC | | 314 | 55 |
| 8 | *E. coli*-987P | | F- CTGCCAGTCTATGCCAAGTG  R- ACGGTGTACCTGCTGAACGAATAG | | 459 | 58 |
