# Supplementary material for: Oral phage therapy with microencapsulated phage A221 against Escherichia coli infections in weaned piglets
Source: BMC Vet Res. 2023 Sep 20;19:165. doi: 10.1186/s12917-023-03724-y (PMC10510151; doi:10.1186/s12917-023-03724-y)

**Fig. S1 PCR amplified of virulence gene** (M, DL2000 DNA Marker;1, *E. coli*-K88;2, *E. coli*-K99;3, *E. coli*-Stx1;4, *E. coli*-Stx2;5, *E. coli*-F18;6, *E. coli*-Stb;7, *E. coli*-LT;8, *E. coli*-987P; -, Negative).


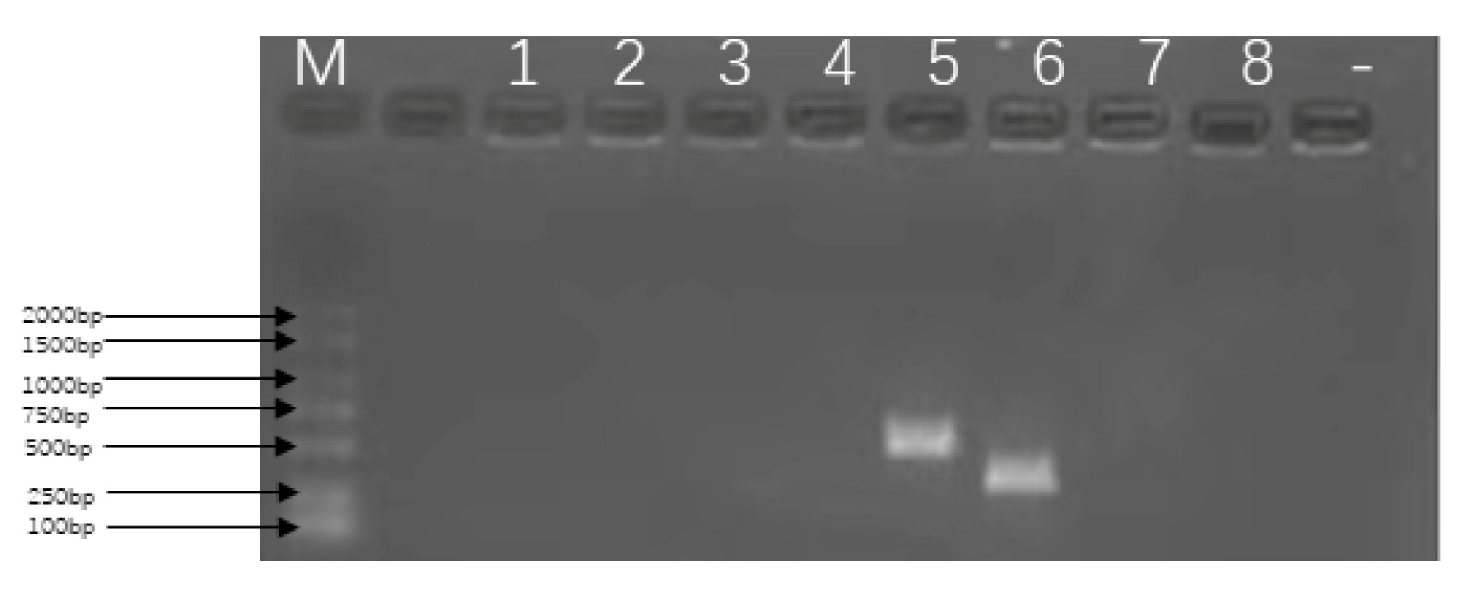

Supplement: Supplementary file 2 — Additional file 2: Fig. S1. PCR amplified of virulence gene (M, DL2000 DNA Marker;1, E. coli-K88;2, E. coli-K99;3, E. coli-Stx1;4, E. coli-Stx2;5, E. coli-F18;6, E. coli-Stb;7, E. coli-LT;8, E. coli-987P; -, Negative). [file 12917_2023_3724_MOESM2_ESM.docx]
